# Influence of maxillary advancement surgery on skeletal and soft-tissue changes in the nose — a retrospective cone-beam computed tomography study

**DOI:** 10.1186/s13005-015-0080-y

**Published:** 2015-07-09

**Authors:** Andreas F. Hellak, Bernhard Kirsten, Michael Schauseil, Rolf Davids, Wolfgang M. Kater, Heike M. Korbmacher-Steiner

**Affiliations:** 1 Department of Orthodontics, University Hospital, Georg-Voigt-Strasse 3, Marburg, 35039 Germany; 2 Private Practice, Bad Homburg, Germany

**Keywords:** Nasal changes, Orthognathic surgery, Retrognathia, CBCT superimposition, Three-dimensional analysis

## Abstract

**Objectives:**

Surgical correction of skeletal maxillary retroposition is often associated with changes in the morphology of the nose. Unwanted alar flaring of the nose is observed in many cases. The aim of the present study was therefore to investigate the influence of surgical advancement of the maxilla on changes in the soft-tissue morphology of the nose. Having a coefficient that allows prediction of change in the nasal width in Caucasian patients after surgery would be helpful for treatment planning.

**Materials and methods:**

All 33 patients included in this retrospective study were of Caucasian descent and had skeletal Class III with maxillary retrognathia. They were all treated with maxillary advancement using a combination of orthodontic and maxillofacial surgery methods. Two cone-beam computed tomography (CBCT) datasets were available for all of the study's participants (16 female, 17 male; age 24.3 ± 10.4 years): the first CBCT imaging was obtained before the planned procedure (T0) and the second 14.1 ± 6.4 months postoperatively (T1). Morphological changes were recorded three-dimensionally using computer-aided methods (Mimics (Materialise NV, Leuven/Belgium), Geomagic (Geomagics, Morrisville/USA)). Statistical analysis was carried out using SPSS 21 for Mac.

**Results:**

The mean sagittal advancement of the maxilla was 5.58 mm. The width of the nose at the alar base (Alb) changed by a mean of + 2.59 mm (±1.26 mm) and at the ala (Al) by a mean of + 3.17 mm (±1.32 mm). Both of these changes were statistically highly significant (*P* = 0.000). The increase in the width of the nose corresponded to approximately half of the maxillary advancement distance in over 80 % of the patients. The nasolabial angle declined by an average of −6.65° (±7.71°).

**Conclusions:**

Maxillary advancement correlates with a distinct morphological change in nasal width. This should be taken into account in the treatment approach and in the information provided to patients.

## Introduction

Changes in the position of the maxilla and/or mandible are associated with corresponding changes in the soft tissue overlying the bone [1]. After surgical correction of maxillary retrognathia with maxillary advancement or bimaxillary surgery, with maxillary advancement and mandibular setback, undesirable changes in the nose have been observed in some cases. For many patients, a disturbing aesthetic appearance is the reason for undergoing surgery, in addition to functional problems [2]. It has been clinically and scientifically proven that the external nose undergoes changes in the context of surgical relocation of the maxilla [3]. This aspect should be examined in greater detail, and it would be of interest to know in what way advancement of the maxilla leads to alar flaring. Measurement of a coefficient capable of relating the skeletal displacement to nasal changes might be helpful for surgical planning and patient education.

The effect of maxillofacial surgery on the facial soft tissue has been investigated in many studies in the past [1, 4–16]. However, there is a lack of research on the relationship between the advancement distance and the amount of alteration measured [10, 17, 16]. A wide variety of analyses have been used for the purpose. The methods most often used in the past have included photography and two-dimensional lateral cephalography [18–21]. Recently, various optical procedures such as laser projection, glancing-light projection, and stereophotogrammetry have made it possible to capture spatial, three-dimensional parameters [18, 22, 23]. In radiography, computed tomography (CT) and cone-beam computed tomography (CBCT) can be used [18, 22–24]. In contrast to optical procedures, radiographic methods are not limited to depicting only the surface of the body; deeper bone structures can also be captured. Three-dimensional changes in the osseous structures and the resulting changes in the soft tissues can be analyzed using CBCT.

Only patients of Asian descent have previously been investigated in connection with this topic [8, 11, 13, 15, 16, 25–27, 14]. Due to ethnic differences in facial structure, it is not possible to transfer the findings to Caucasian patients [26]. It is regarded as clinically and scientifically proven that the external nose is subject to flattening and widening when surgical repositioning is carried out in the maxilla.

The aim of the present study was therefore to use three-dimensional CBCT data to detect dependencies between skeletal advancement of the maxilla and alterations in the morphology of the nose. In the case of a confirmed association, the aim was to evaluate whether any dependency on the extent of the advancement could be identified.

## Materials and methods

Two CBCT datasets for each of 33 patients (16 female, 17 male) — i.e., a total of 66 CBCTs — were examined retrospectively. The patients’ mean age was 24.3 ± 10.4 years. All of the patients had an Angle class III anomaly with maxillary retrognathia preoperatively. They were examined clinically and radiographically in the Department of Oral and Maxillofacial Surgery in Bad Homburg, Germany, and were of Caucasian descent.

Weight variations of more than 5 kg were not permitted during the study period. This information was obtained from the anesthesia protocol. The following inclusion and exclusion criteria were set. The criteria for inclusion in the patient group were:

- Caucasian descent
- Maxillary retrognathia (SNA < 80°)
- Surgical advancement of the maxilla
- During the preoperatively conducted model operation, available current plaster jaw models had to allow stable occlusion in Angle class I

The criteria for exclusion from the patient group were:

- No maxillary retrognathia (SNA > 80°)
- Not of Caucasian descent
- Additional intraoperative augmentation of the midface
- Craniofacial anomalies or syndromes, or any form of cheilognathouranoschisis

Surgically, a Le Fort I osteotomy of the maxilla in combination with bridle sutures for the bases of the two ala was used [16, 17, 28, 29]. The Le Fort I osteotomy method used by the surgeon (exclusively W.K.) is based on the fracture line described by René Le Fort in 1901 [30]. The osteotomy starts at the piriform aperture cranial to the anterior nasal spine and passes through the facial maxillary sinus wall, the zygomaticoalveolar crest, and the maxillary tuberosity to the dorsal surface of the maxillary sinus, separates the caudal tip of the pterygoid process of the sphenoid bone, bends forward to the nasal cavity, runs through the lateral nasal wall in its basal portion, and from there returns to the piriform aperture [30]. After repositioning of the maxilla using the face-bow and glabella support, or with a surgical splint prepared in advance to determine the occlusal relationship of the maxilla to the mandible, the maxilla is fixed in its final position using an adapted titanium mini-plate and accompanying screws. In addition, alar cinch sutures are created for the bases of the two nostrils and attached with this technique at the anterior nasal spine [17, 3, 29, 28]

All 33 patients underwent maxillary advancement. A bimaxillary operation with maxillary advancement and mandibular setback was carried out in the majority of the patients. Table 1 presents an overview of the additional surgical procedures used and their frequencies.

**Table 1:** All patients (*n* = 33) underwent maxillary advancement

Mandibular setback	Mandibular advancement	Maxillary dorsal impaction	Maxillary impaction	Maxillary repositioning as two-piece maxilla	Rotation of the maxilla	n	%
✓	✗	✗	✓	✗	✗	14	42 %
✓	✗	✗	✗	✗	✗	7	21 %
✓	✗	✓	✗	✗	✗	3	9 %
✗	✗	✗	✗	✗	✗	3	9 %
✓	✗	✓	✗	✓	✗	2	6 %
✗	✗	✓	✗	✗	✗	2	6 %
✗	✓	✗	✗	✗	✓	1	3 %
✗	✗	✗	✓	✗	✗	1	3 %

The table shows the distribution of additional surgical procedures (in numbers and percentage distribution)

The first CBCT imaging procedure was carried out 2–3 weeks before the planned procedure (T0). The second images were obtained after surgery (T1; 14.1 ± 6.4 months postoperatively), but not before the completion of soft-tissue healing. Completion of soft-tissue healing was defined as 6 months after surgery, based on the results of earlier studies [31, 32].

Identical parameters were used for all CBCT imaging procedures. All of the CBCT images were taken with a KaVo 3D eXam device (KaVo Dental Ltd., Bieberach/Riss, Germany). This CBCT device has a high-frequency X-ray source with a constant potential of 120 kVp (kilovolt peak) and pulsed 3–8 mA. The settings used for all of the CBCT imaging procedures were identical, with a scanning time of 26.9 s, a voxel size of 0.25 × 0.25 × 0.25 mm, an effective irradiation period of 7 s, anode voltage of 120 kV, and tube current of 5 mA (for details, see KaVo). The maximum field of view (FOV) of the device was 16 × 13 cm. Depending on the issue and indication, the height of the FOV was 6, 8, or 11 cm and the image had to include all relevant points.

All of the patients provided written informed consent to the inclusion of their data in the study. The data were pseudonymized. The CBCT datasets were given identifiers numbered 1–66 and the underlying names of the patients were deleted. Deallocation was only permissible for the director of the study (HKS).

Collection and analysis of the soft-tissue datasets were carried out using the Mimics 15.0 (Materialise NV, Leuven/Belgium) computer program. Table 2 shows all of the relevant points, distances, planes, and angles.

**Table 2:** Relevant points, distances, planes and angles

Variable	Explanation
Al	Alar width
Alb	Alar base width
Albl	Deepest point at the transition between the left ala to the cheek to air at the sagittal level (left alar base)
Albr	Deepest point at the transition between the right ala to the cheek to air at the sagittal level (right alar base)
All	Furthest transverse extent of the left ala
Alr	Furthest transverse extent of the right ala
Co	Columella tangent point, bridge of the nose
FH	Frankfurt horizontal plane, two poria and an infraorbital point
Ls	Labrale superius, edge of the upper lip (transition from vermilion border to white portion)
Sn	Subnasal point, transition from the bridge of the nose to the upper lip
Sn–Ls–Co	Nasolabial angle

**Fig. 1:**
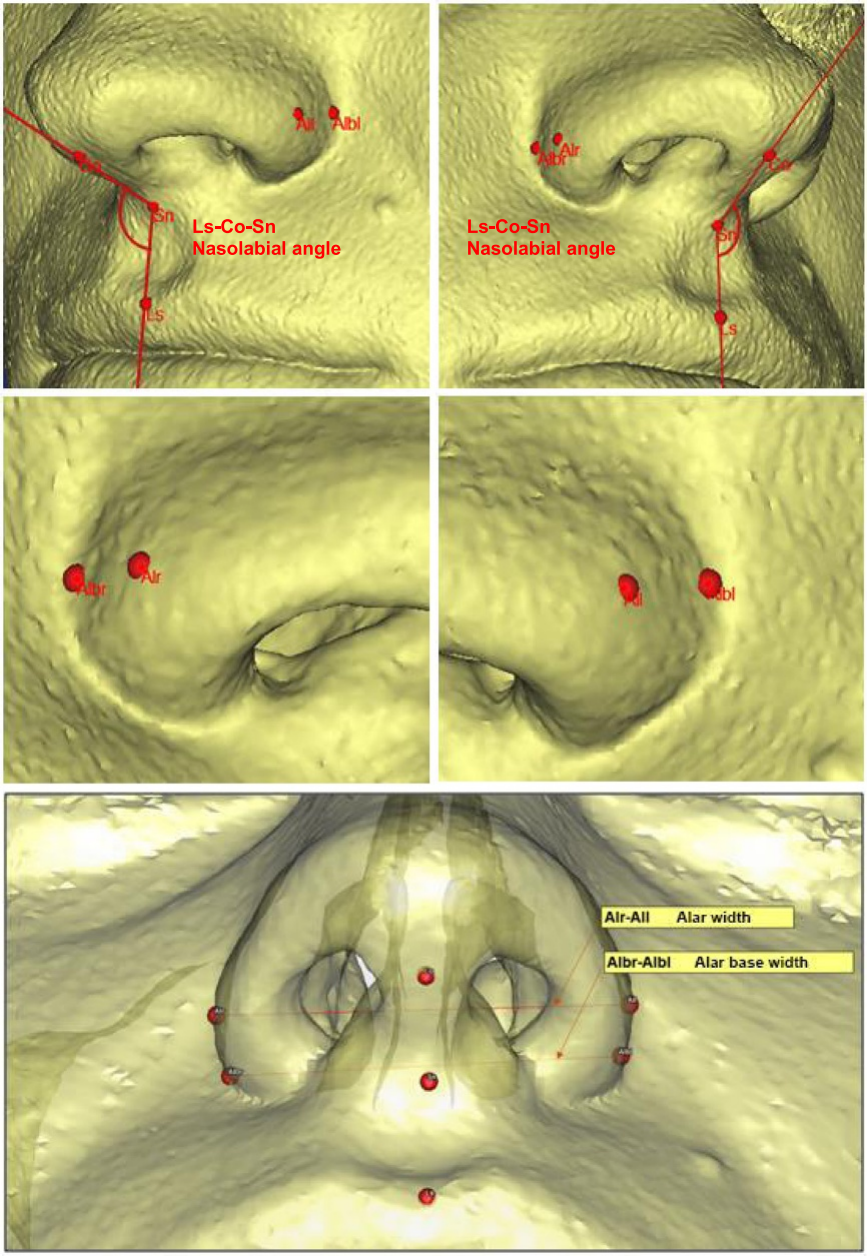
Soft-tissue points and distances. The nasolabial angle (NLA) is the angle between the labrale superius (Ls), the columellar tangential point (Co), and the subnasal point (Sn). The alar base width (Alb) is the distance from the alar base on the right (Albr) to the alar base on the left (Albl). The alar width (Al) is the distance from the right ala (Alr) to the left ala (All)

The nasolabial angle (NLA [33]), alar base width (Alb), and alar width (Al) were measured to assess changes in the nasal soft tissues. The soft-tissue points shown in Fig. 1 were used for measurements. The following distances and angles were formed from the measurement points:

- Alar base width (Alb): from Albr to Albl: Alb distance
- Alar width (Al): from All to Alr: Al distance
- Nasolabial angle (NLA): angle between Ls to Co and Sn

**Fig. 2:**
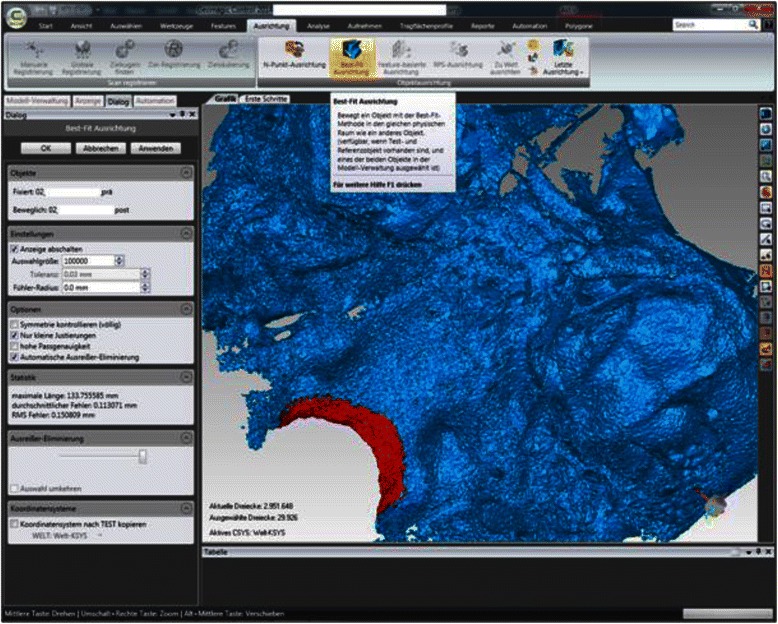
Superimposition at the foramen magnum with the surrounding bone (red points) using Geomagic Control

**Fig. 3:**
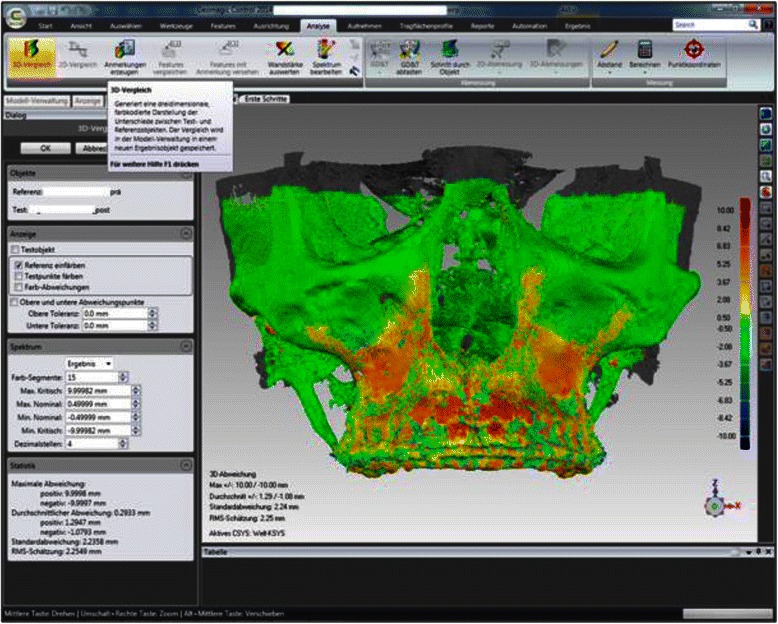
The user interface in Geomagic Control after completion of the superimposition of the cone-beam computed tomograms from T0 and T1. The colors diverge from green to show the skeletal changes

**Fig. 4:**
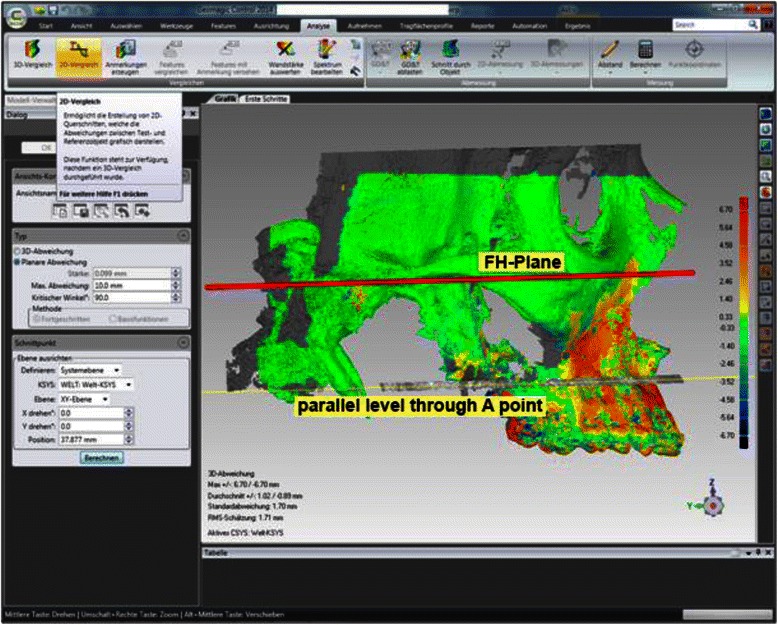
Visualization of sagittal repositioning. The figure shows a level parallel to the Frankfurt plane placed through the A point

**Fig. 5:**
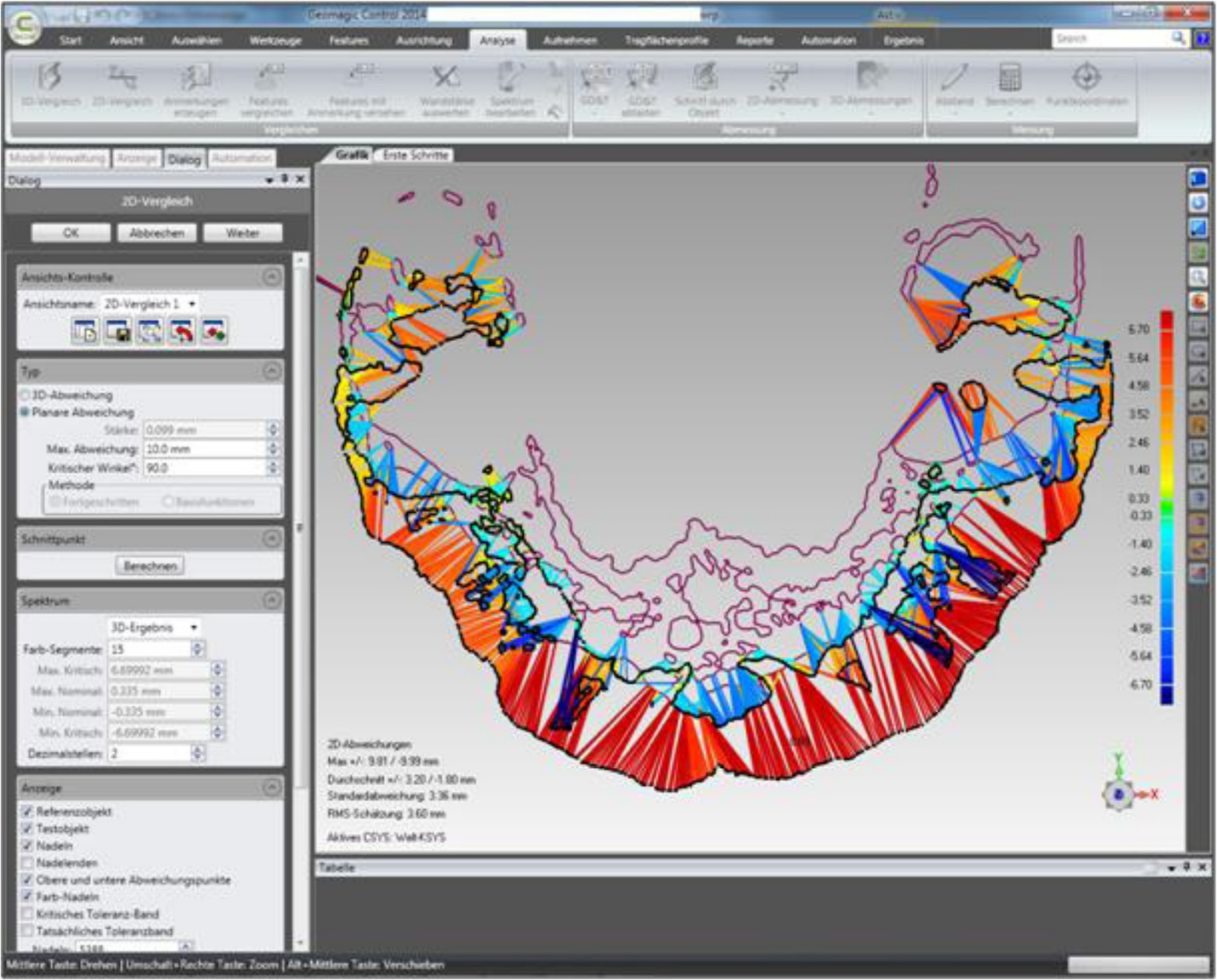
Visualization of sagittal repositioning (mm). The measurement values were collected in regions 13, 11, 21, and 23 in the needle view (red needles). The repositioning of these points that is calculated represents the skeletal advancement of the maxilla

For measurement of the skeletal repositioning of the maxilla, the CBCT for T0 and T1 were superimposed using Geomagic Control 2014.0 (Geomagics, Morrisville, USA). Superimposition was carried out at the foramen magnum with surrounding bone and at the anterior skull base at 100,000 polygons [34, 35] (Fig. 2). Figure 3 shows the user interface in Geomagic Control after completion of the superimposition. A level parallel with the Frankfurt plane was placed through the A point (Fig. 4). At this level, individual measurement values were collected in regions 13, 11, 21, and 23 in the needle view, and a mean was calculated (Fig. 5). The calculated repositioning of these points represents the skeletal advancement of the maxilla. All of the measurements were repeated by the same operator after an interval of 2 weeks.

### Statistics

Statistical analysis was performed using IBM SPSS Statistics for Mac, version 21.0 (IBM Corporation, Armonk, New York, USA). Methodological error was estimated using Spearman rank correlation. As the Shapiro-Wilk test showed significant deviations from the normal distribution, Student’s *t*-test and the Wilcoxon test were carried out. The significance level was set at *P* < 0.05.

Using SPSS, a formula was generated to calculate the amount of change in the width of the nose. A regression between the amount of maxillary advancement and the widening of the ala and alar base was prepared by transformation of the sagittal displacement distance with the square root and soft-tissue enlargement with the logarithm. The transformed variables followed a sufficiently Gaussian normal distribution. The relation of the transformed values was not purely linear, but curved. A second-degree polynomial was used to calculate the regression.

## Results

The reproducibility of the measurement values represented by Spearman rank correlation showed highly significant correlations (*P* = 0.000) (Tables 3). The mean sagittal repositioning of the maxilla was 5.58 mm. The calculated smallest repositioning distance was 2.02 mm and the largest distance was 10.84 mm (Table 4).

**Table 3:** Measurement error represented by correlation between the first measurement and follow-up measurement of nasal soft-tissue changes (Alb, Al, Sn–Ls–Co) and maxillary advancement (M–A); intraoperator correlation

		*r*	*P*
Nasal soft-tissue changes before treatment	Alb	0.9987	<0.000005***
	Al	0.9980	<0.000005***
	Sn–Ls–Co	0.9766_Sp_	<0.000005***
Nasal soft-tissue changes after treatment	Alb	0.9978	<0.000005***
	Al	0.9976	<0.000005***
	Sn–Ls–Co	0.9729_Sp_	<0.000005***
Maxillary advancement	M–A	0.9566	<0.000005***

*r* and *P* from the product–moment correlation or from Spearman rank correlation (Sp) (*n* = 33) for alar base, alar width, and nasolabial angle

***p< 0.001

**Table 4:** Skeletal maxillary advancement (M–A) on the Frankfurt plane (mm)

Region	n	Mean	SD	Maxillary advancement (mm)			Minimum	Maximum
				Mean	68 % CI			
Front	33	5.580	2.412	5.728	2.820	8.159	2.0163	10.841

*CI* confidence intervals, *SD* standard deviation

The width of the nose increased highly significantly between T0 and T1 as a result of the maxillary advancement (*P* = 0.000). As Table 5 shows, highly significant changes in the alae (Al), alar base (Alb), and nasolabial angle (NLA) were observed (*P* = 0.000). The alar width Al (mean + 3.17 ± 1.32 mm) increased in all 33 patients. The alar base width Alb (mean + 2.59 ± 1.26 mm) also increased. The nasolabial angle declined in 28 patients and increased in five patients (mean −6.65° ± 7.71°). As Table 6 shows, the increases in the width of the alar base and alar width correlated highly significantly with the skeletal advancement of the maxilla (*P* = 0.000).

**Table 5:** Comparison of values at T0 and T1; *P* with Student’s *t*-test and the Wilcoxon test (W) (*n* = 33)

		Mean	SD	Increase		*P*
				Mean	SD	
Alb	Before treatment	33.665	2.632	3.171	1.322	<0.000005***
	After treatment	36.837	2.651			
Al	Before treatment	35.459	2.912	2.588	1.255	<0.000005***
	After treatment	38.047	2.851			
Sn–Ls–Co	Before treatment	102.992	14.388	−6.652	7.712	0.00002***W
	After treatment	96.339	12.748			

***p< 0.001

**Table 6:** Correlations of changes in the external nose (alar base width, alar width, nasolabial angle) relative to skeletal advancement of the maxilla

Increase in	Region	n	rho	*P*
Alar base width	Front	33	0.6949	0.00001***
Alar width	Front	33	0.7688	<0.000005***
Nasolabial angle	Front	33	−0.3102	0.079

Soft-tissue parameters as the difference in means from T0 to T1; skeletal measurements as means from T1 to T0 for maxillary advancement. *P* from Spearman’s rank correlation

***p< 0.001

The change in the nasolabial angle was not entirely independent of the sagittal repositioning, but this was not statistically significant (*P* > 0.05).

Based on the results, the following formulas were developed to allow prediction of soft-tissue changes relative to skeletal advancement:

Alar base width:

- Patients (*n* = 33), regression coefficient *R*^2^ = 0.4764, standard deviation *s* = 0.1338959
- Formula for y = ∆Alb in the course of x = maxillary advancement:
  - log(ΔAlb) = 0.4558889 + 0.124167 × z + 0.010571 × z^2^, where z = (√(maxillary advancement) – 2.305)/0.5255

Alar width:

- Patients (*n* = 33), regression coefficient *R*^*2*^ = 0.5281, standard deviation *s* = 0.1403169
- Formula for y = ∆Alin the course of x = maxillary advancement:
  - log (ΔAl) = 0.3704371 + 0.1435941 × z − 0.001474 × z^2^, where z = (√(maxillary advancement) – 2.305) / 0.5255

Advancement by 5 mm would thus lead to a mean widening of the alar base of ≈ 2.8 mm. Ninety percent of the patients would have widening of between ≈ 1.7 mm and ≈ 4.6 mm (Table 7). As Table 8 shows, advancement of 5 mm would lead to a mean widening of the ala of ≈ 2.2 mm. Ninety percent of the patients could expect widening of between ≈ 1.3 mm and ≈ 3.8 mm.

**Table 7:** Prediction of changes in alar base width

Maxillary advancement	Percentiles for increase in Alb (mm)				
	5.0 %	10.0 %	50.0 %	90.0 %	95.0 %
2 mm	1.13653	1.26409	1.88730	2.81777	3.13401
3 mm	1.29681	1.44235	2.15346	3.21515	3.57598
4 mm	1.46956	1.63449	2.44031	3.64343	4.05233
5 mm	1.65802	1.84409	**2.75326**	4.11066	4.57200
6 mm	1.86481	2.07410	3.09666	4.62337	5.14224
7 mm	2.09245	2.32728	3.47467	5.18774	5.76995
8 mm	2.34348	2.60649	3.89153	5.81012	6.46218
9 mm	2.62062	2.91473	4.35173	6.49721	7.22638
10 mm	2.92678	3.25524	4.86013	7.25626	8.07062

Approximate percentiles for Alb relative to maxillary advancement. A advancement of 5mm would lead to a mean widening of the alar base width of 2.8 mm

**Table 8:** Prediction of changes in alar width

Maxillary advancement	Percentiles for increase in Al (mm)				
	5.0 %	10.0 %	50.0 %	90.0 %	95.0 %
2 mm	0.77994	0.87191	1.32704	2.01974	2.25791
3 mm	0.95805	1.07102	1.63009	2.48098	2.77353
4 mm	1.13726	1.27136	1.93499	2.94504	3.29231
5 mm	1.32078	1.47652	**2.24725**	3.42030	3.82361
6 mm	1.51028	1.68837	2.56968	3.91103	4.37221
7 mm	1.70677	1.90802	2.90400	4.41985	4.94103
8 mm	1.91093	2.13626	3.25137	4.94855	5.53207
9 mm	2.12328	2.37365	3.61268	5.49846	6.14682
10 mm	2.34424	2.62067	3.98863	6.07066	6.78649

Approximate percentiles for Al relative to maxillary advancement. A advancement of 5mm would lead to a mean widening of the alar width of 2.2 mm

## Discussion

Investigations have been carried out since the 1980s to analyze associations between changes occurring in the facial soft tissue in connection with Le Fort I osteotomies in dysgnathia surgery. A correlation between sagittal repositioning of the maxilla and the amount of alar flaring was not identified at that time [36]. Preoperative and postoperative lateral cephalograms or photographs were used for measurements in the early studies [10]. However, these techniques did not allow any conclusions to be drawn regarding correlations with the changing width of the nose [11].

Three-dimensional measurement of hard-tissue models obtained from CBCT and CT imaging is now increasingly being used, mainly for the planning of dental implants in dental medicine [37]. The amount of correlation between the initial measurements and control measurements of the soft tissue after 2 weeks is highly significant. Discussion is needed on mistakes during the reproducibility of these measurements. The reproducibility and reliability of different measurement points on lateral cephalograms has been frequently investigated in the past and has also been classified [38–40]. Similar findings were obtained with measurement points derived from lateral cephalograms created with CBCT [41]. The measurement points created on three-dimensional surfaces using CBCT scans are thus regarded as being highly reliable [42–44].

The sagittal movement of the maxilla was registered on a plane parallel to the Frankfurt plane. In 2012, Daboul et al. reported that the reproducibility and reliability of the Frankfurt plane on 3D multiplanar reformatting (MPR) images was excellent [45]. Similar results were described by Ludlow et al. in 2009, who found amongst other things that identification of cephalometric landmarks is significantly more precise with MPR views of CBCT landmarks [46].

The first analysis of soft-tissue areas on the basis of three-dimensional CBCT reconstructions was reported by Han et al. in 2005. However, the patients included in the study were all of Asian descent [25]. In 2010, Kim et al. also investigated 3D reconstructions from CBCT images after repositioning of the maxilla at the Le Fort I level [47]. However, transverse changes in the size of the nose were not investigated. In 2012, the topic was again addressed by Park et al. using a new method, and transverse changes in the size of the nose were measured. The study also only included patients of Asian descent [14]. Farkas et al. noted that Asian individuals tend to have a nasal morphology that is very different from that in Caucasians [26, 48]. Direct comparison thus does not appear useful. In contrast to other publications, the present study therefore only includes patients of Caucasian descent and exclusively patients with maxillary retrognathic position. The results of the measurements of the width of the nose before surgical intervention were also compared with the results presented by Farkas. The T0 results were very similar to those of Farkas et al. in relation to the anthropometric measurements of Caucasian noses.

Measurement of soft-tissue models obtained from CBCTor CT imaging using Mimics has been confirmed as a valid method in various studies [49, 50]. Correlation of the initial measurements and follow-up measurements in the present study also showed that the measurement method is extremely accurate.

Using 3D radiographic evaluation, the present study shows that the transverse size of the nose increases when the maxilla is advanced during Class III surgical correction of occlusion anomalies. This result is also supported by the 3D photographic studies reported by Honrado et al. in 2006 [5]. A considerable disadvantage with the use of methods based on conventional light is that only the skin is detected as a surface, with no information about the underlying structures. In addition, the undercutting (“shadow”) that is produced by conventional light during detection of a three-dimensional surface can make evaluation impossible in some areas [23, 24]. These problems can be avoided with three-dimensional radiographic methods such as CBCT. Thanks to the different physical properties of skin and bone, the skin remains in the field of view when the underlying bone is being examined. In addition, CBCT produces an image that is true to scale, and undercutting does not occur.

The use of ionizing radiation for evaluation of a problem that is primarily aesthetic in nature may be questioned. It should be noted here that there is no indication for CBCT in the evaluation of soft tissue. Instead, when CBCT is required in any case for another indication, its findings can be enhanced using the techniques described here.

Overall, the results appear to be of major importance for everyday clinical purposes, since according to Göz et al., a poor aesthetic appearance is the most important reason why patients decide to undergo surgery for dysgnathia [2]. Subsequent widening of the nose is often regarded as an undesirable aesthetic change [2, 51]. If functional aspects allow it, the initial shape of the nose and the amount of maxillary advancement should be taken into account during the planning of the operation [27, 52, 28].

In addition to the usual information provided before surgery, it should therefore also be drawn to the patient’s attention that the morphology of the external nose changes postoperatively and that surgical correction of the nose may become necessary later on [27, 52, 53]. The three-dimensional alteration coefficient of approximately 50 % calculated in the present study (with 1 mm of sagittal advancement of the maxilla equaling an increase in the width of the nose by 0.5 mm) could be used for preoperative assessment of the potential change.

The nasolabial angle decreases in most cases after sagittal repositioning, but there was no statistically significant correlation.

## Conclusions

Maxillary advancement has effects on nasal morphology in individuals of Caucasian descent. The widening of the nose and narrowing of the nasolabial angle demonstrated in the present study may have a negative influence on the postoperative aesthetic result and should therefore be taken into account both in treatment planning and also in the information provided to patients. The correlation coefficient calculated between sagittal advancement and soft-tissue changes in the nose may make prediction easier.

### Ethical considerations

The study was compiled with the rules laid down by the Declaration of Helsinki. It was explained to the patients that inclusion of their data in the study was voluntary and that confidentiality and anonymity were guaranteed. They were also able to withdraw from the study at any time before publication without needing to give any reason. Written informed consent was obtained from all of the participants.

## Abbreviations

Al: Alar width
Alb: Alar base width
Albl: Alar base on the left
Albr: Alar base on the right
All: Left ala
Alr: Right ala
CBCT: Cone-beam computed tomography
Co: Columellar tangential point
CT: Computed tomography
FOV: Field of view
Ls: Labrale superius
MPR: Multiplanar reformatting
NLA: Nasolabial angle
Sn: Subnasal point
SNA: Sella-nasion-A point angle
